# Randomised controlled non-inferiority trial of primary care based facilitated access to an alcohol reduction website (EFAR-FVG)

**DOI:** 10.1186/1940-0640-8-S1-A83

**Published:** 2013-09-04

**Authors:** Paul Wallace, Piero Struzzo, Roberto della Vedova, Costanza Tersar, Lisa Verbano, Harris Lygidakis, Richard MacGregor, Nick Freemantle, Emanuele Scafato

**Affiliations:** 1 National Institute for Health Research, Primary Care Research Network, London, UK; 2 Regional Centre for the Training in Primary Care, Monfalcone, Italy; 3 Movimento Giotto, Bologna, Italy UK; 4 Codeface Ltd, Hove, UK; 5 Department of Primary Care and Population Health, University College London, London, UK; 6 National Observatory on Alcohol, Center for Epidemiology, Surveillance and Health Promotion, National Institute of Health (ISS), Rome, Italy

## Introduction

There is a strong body of evidence demonstrating effectiveness of brief interventions by primary care professionals for risky drinkers but implementation levels remain low. Facilitated access to an alcohol reduction website constitutes an innovative approach to brief intervention, offering a time-saving alternative to face to face intervention, but it is not known whether it is as effective.

## Objective

To determine whether facilitated access to an alcohol reduction website is equivalent to face to face intervention.

## Methods

Randomised controlled non-inferiority trial for risky drinkers comparing facilitated access to a dedicated website with face to face brief intervention conducted in primary care settings in the Region of Friuli Venezia-Giulia, Italy. Adult patients are given a leaflet inviting them to log on to a website to complete the AUDIT-C alcohol screening questionnaire. Screen positives are requested to complete an online trial module including consent, baseline assessment and randomisation to either standard intervention by the practitioner or facilitated access to an alcohol reduction website. Follow up assessment of risky drinking is undertaken online at 1 month, 3 months and 1 year using the full AUDIT questionnaire. Proportions of risky drinkers in each group will be calculated and non-inferiority assessed against a specified margin of 10%. The trial is being undertaken as an initial pilot and a subsequent main trial.

## Results

12 practices have participated in the pilot, and more than 1300 leaflets have been distributed. 89 patients have been recruited to the trial with a one month follow-up rate of 79%.

## Discussion

The findings of the pilot study suggest that the trial design is feasible, though modifications will be made to optimize performance in the main trial which will commence in January 2014. Plans are concurrently underway to replicate the trial in Australia, and potentially in the UK and Spain.

